# Optimization of ultrasound‐assisted extraction by response surface methodology, antioxidant capacity, and tyrosinase inhibitory activity of anthocyanins from red rice bran

**DOI:** 10.1002/fsn3.1371

**Published:** 2020-01-05

**Authors:** Yujie Wang, Lei Zhao, Ruoyu Zhang, Xiushi Yang, Yanghua Sun, Longlong Shi, Peng Xue

**Affiliations:** ^1^ School of Public Health and Management Weifang Medical University Weifang China; ^2^ Institute of Crop Sciences Chinese Academy of Agricultural Sciences Beijing China

**Keywords:** anthocyanins, antioxidant activity, red rice bran, response surface methodology, tyrosinase inhibitory activity

## Abstract

The anthocyanins contents from red rice bran were characterized by HPLC/MS. Response surface methodology was used to optimize the ultrasound‐assisted extraction of red rice bran anthocyanin. The antioxidant activities were evaluated in terms of IC_50_. The tyrosinase inhibitory activities of the anthocyanin samples from red rice bran and the standard substances were determined by a spectrophotometric method. According to mass spectrometry information, the main component of anthocyanins is paeoniflorin (*m/z* = 480). The optimized anthocyanin level was 5.80 mg/g under the following conditions: solid–liquid ratio of 1:17.46; ethanol concentration of 78.37%; ultrasonication time of 55.23 min; and pH of 2.31. The IC_50_ value of the DPPH radical scavenging and the superoxide anion scavenging activities of the sample were 53.51 and 2,375 μg/ml; those of the standard were 14.60 and 64.74 μg/ml; and those of vitamin C were 24.45 and 136.25 μg/ml, respectively. The IC_50_ values of the tyrosinase inhibition activities of the sample and Vc were 4.26 and 2.18 μg/ml, respectively. There is a significant difference (*p* < .05) between the activities of the three, which may be caused by the purity of the extract. Red rice bran anthocyanins have valuable research and development prospects as skin whiteners and healthcare products.

## Graphical abstract

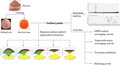

## 1 INTRODUCTION

As chemical methods have developed over the past century, various artificial pigments have been manufactured and applied to the food industry and cosmetics. Artificial pigments are made from aniline dye which was separated from coal tar. A large number of studies have shown that artificial pigments do not provide any nutrients to the human body (Deng et al., 2013; Valentina & Rita, 2017; Zhu, 2018). Because of their potential carcinogenic, teratogenic, and mutagenic activities (Boo et al., 2012), the use of artificial pigments has been gradually reduced. Therefore, the development of natural pigments with almost no toxic side effects is urgently needed to replace artificial colors.

Anthocyanins are flavonoid polyphenolic compounds based on the C6‐C3‐C6 molecular backbone (Abdel‐Aal, Young, & Rabalski, 2006; Chen, Choi, Kozukue, Kim, & Friedman, 2012). Anthocyanins are natural water‐soluble pigments that present different colors ranging from red and purple to blue and widely found in the flowers, fruits, stems, leaves, and roots of plants. Importantly, anthocyanins can be as additives not only for dyeing foods and cosmetics but also provide a variety of biological activities. Anthocyanins have been proven to inhibit tyrosinase activity and prevent diabetes (Chen, Choi, et al., 2012). Therefore, anthocyanins are beneficial substances to human health. Currently, many studies have examined the anthocyanins in fruits and vegetables (Boo et al., 2012; Fujiwara, Kono, Ito, & Ito, 2018; Homoki et al., 2016; Liu, Mu, Sun, Zhang, & Chen, 2013), but limited research has investigated the anthocyanins in grains. Colored rice, such as black rice and red rice, is rich in anthocyanins (Hosoda et al., 2018). Red rice which originated in China and has a history of over 1,000 years of use is a potential raw material for extracting anthocyanins. Studies have shown that high anthocyanin levels of approximately 7.9–34.4 mg/100 g are present in unpolished red rice (Hirawan, Diehljones, & Beta, 2011; Min, Mcclung, & Chen, 2011), while they are barely detectable in polished red rice. This finding indicates that anthocyanins are mainly found in the red rice bran. However, in the production, processing, and application of red rice, bran are not fully utilized. Only a small fraction of red rice bran is used to make whole grain foods or to raise livestock. Most of the bran is burned and used as a fertilizer, representing a large waste of resources. The anthocyanins extracted from red rice bran are desirable for making cosmetics because of their antioxidant activity and ability to inhibit tyrosinase activity. They also can be used in the food industry as a natural pigment additive.

Several of the most common approaches to extracting anthocyanins are solvent extraction, microwave‐assisted extraction, ultrasound‐assisted extraction, biological enzymatic hydrolysis, and supercritical fluid extraction (Boo et al., 2012; López et al., 2018). In recent years, ultrasonic‐assisted extraction has achieved high recovery rates (Ding et al., 2018), and this method has been widely used in the extraction of plant polyphenols. The extraction process of anthocyanins consumes large amounts of water, energy, and organic solvents that are harmful to the environment and users (Liu, Mu, et al., 2013). Recent trends have paid more attention to sustainable development and reduction in the environmental impact of energy, water, and material consumption. The conditions of ultrasonic‐assisted extraction need to be optimized. Response surface methodology (RSM) is an effective statistical tool that allows for the simultaneous optimization of multiple variables. This method can predict the best performance conditions with a minimum number of experiments (Jiang et al.,^2015^) and is widely applied for optimizing conditions in the food industry now.

In the present study, the raw materials consisted of the bran produced during the processing of the red rice raw grain. RSM was used to optimize the experimental conditions of the ultrasonic‐assisted extraction of anthocyanins. The antioxidant capacity of the anthocyanin extract and its inhibitory effect on tyrosinase were preliminarily studied. This study provides the basis for the future application of red rice bran anthocyanins in the medical, cosmetic, and health industries, where they can be used to make healthy foods and natural pigments.

## 2 MATERIALS AND METHODS

### 2.1 Chemicals

Red rice bran samples were purchased from Yuanyang County Grain Supply and Marketing Co., Ltd. Paeoniflorin, tyrosinase, and ascorbic acid were purchased from Sigma. Potassium ferricyanide, DPPH, EDTA, pyrogallol (PR), and dithiothreitol (DTT) were purchased from J & K. Acetonitrile and methanol were obtained from Merck. The other chemicals were of analytical grade.

### 2.2 Sample preparation

**Figure 1:**
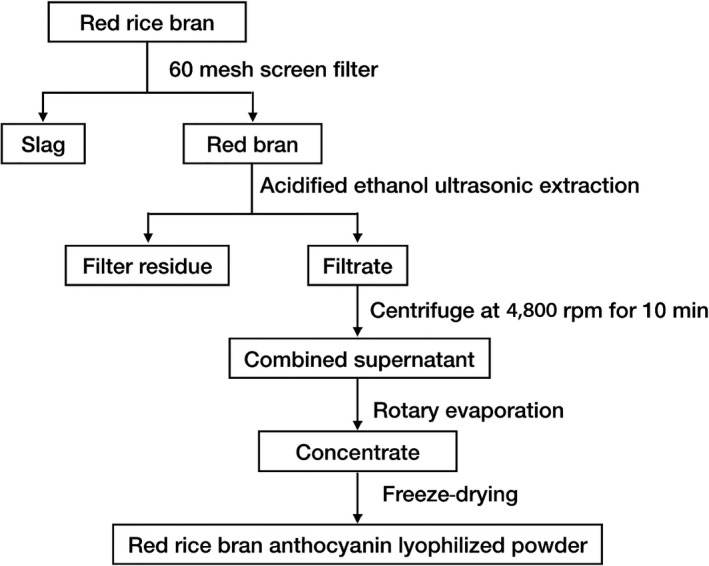
Flowchart of extraction process of red rice bran anthocyanins

The red rice bran was obtained from Yuanyang County Grain Purchase and Sale Co., Ltd.. This bran was ground into powder by a pulverizer and filtered through a 60‐mesh sieve. The selected bran samples were soaked in a 10× volume of acidified ethanol. Extraction was performed using an ultrasound device with a power of 400 W and a frequency of 24 kHz. The filtrate was centrifuged for 10 min at a speed of 4120*g*, concentrated by rotary evaporation, and then freeze‐dried in darkness (Huiqin, Ying, & Hui, 2014). The obtained powder was the lyophilized powder of red rice bran anthocyanins (Figure 1). Water extraction method is carried out according to the above conditions. The obtained water extract and alcohol extract were dissolved in water and methanol at a concentration of 1 mg/ml analyzed by HPLC/MS, respectively.

### 2.3 HPLC/MS conditions

The electrospray ionization (ESI) mass spectrometry (MS) data were recorded on an Agilent‐LC‐1100 instrument with ZORBAX SB‐C18 (100 mm × 3 mm, 1.8 μm) (Agilent). The HPLC conditions for the HPLC‐ESI‐MS analysis were as follows: column temperature: 40°C; injection volume: 20 μl; mobile phase A: 0.1% formic acid aqueous solution; mobile phase B: methanol; gradient elution (mobile phase B concentration): 0 ~ 5 min； 10%, 5 ~ 20 min: 10 ~ 20%, 20 ~ 45 min: 60 ~ 80%, 45 ~ 49 min: 80 ~ 10%, 50 ~ 55 min: 10%, and the flow rate is 0.3 ml/min. The ESI parameters were as follows: the collision gas (N_2_) flow rate was maintained at 10 ml/min, the column oven was 25°C, data were acquired in positive ion mode [M + H]^+^, scans were conducted over *m/z* 50–2000, the spray voltage was 4.5 kV, the capillary voltage was 10 V, and the capillary temperature was 250°C.

### 2.4 Experimental design

Response surface methodology was applied to determine the optimal conditions for anthocyanin extraction. A Box–Behnken experimental design was performed with four independent variables (*X*_1_, solid–liquid ratio; *X*_2_, ethanol concentration; *X*_3_, ultrasonication time; *X*_4_, pH) set at three variation levels. These values were based on the results of single‐factor experiments (Figure 3). The entire experimental design contained 29 experiments; four of them were repetitions of the center point. All experiments were randomly performed to reduce the impact of systematic errors on the results.

### 2.5 Anthocyanin yield

The anthocyanin content was determined by spectrophotometry. The absorbance of the supernatant was measured at 300–700 nm, as mentioned in step 2.2, to determine the maximum absorption wavelength of the red rice bran extract. Paeoniflorin was used as a standard (Klavins, Kviesis, Nakurte, & Klavins, 2018). Methanol was used as the solvent, and the standard solution was prepared by the double dilution method (100–3.125 μg/ml). The absorbance was measured at the maximum absorption wavelength of the extract. Then, using the standard concentration as the abscissa and the absorbance as the ordinate, the standard curve was calculated, and the regression equation was obtained. The total anthocyanin content was expressed as mg of paeoniflorin equivalent to g of freeze‐dried powder as in the following equation:

$$Y=\frac{n\times c\times V}{1000\times m}$$

where Y is the total anthocyanin content, n is the dilution factor, *c* is the anthocyanin concentration of the extract calculated by the regression equation (μg/ml), *V* is the extract volume (ml), and m is the freeze‐dried powder quality (g). Three sets of parallel samples were analyzed for each experiment.

### 2.6 Antioxidant capacity

The antioxidant capacity of red rice bran anthocyanins was evaluated by measuring reducing power, DPPH oxygen free radical scavenging ability, and superoxide anion radical scavenging ability.

#### 2.6.1 Determination of reducing power

This experiment was performed according to Zhang's method (Zhang & Yang, 2005) with minor changes. The sample was dissolved in 0.2 mol/L phosphate buffer at pH 7.4. Aliquots (0.5 ml) of sample solution (1–100 μg/ml) were mixed with an equal volume of 1% potassium ferricyanide solution and incubated in a 50°C water bath for 20 min. A total of 0.5 ml of 10% trichloroacetic acid was added and mixed, and the solutions were diluted with distilled water. Finally, 0.3 ml of ferric chloride was added. After standing for 2 min, the supernatant (100 μl) of each sample was added to a 96‐well plate, and the respective absorbances were recorded at 700 nm with a spectrophotometer. Ascorbic acid (1–100 μg/ml) and anthocyanin standards (0.1–10 μg/ml) were used as positive controls, and distilled water was used instead of the sample solution as a negative control. The tests were performed in triplicate. The higher the absorbance value is, the stronger the reduction ability.

#### 2.6.2 DPPH radical scavenging activity

The antioxidant activity of the anthocyanins was assessed by the DPPH radical scavenging activity assay reported in Zhang's paper (Zhang & Yang, 2005). Samples were prepared in distilled water to different concentrations (1–100 μg/ml), and the reagent and sample were added to the test tube in accordance with Table 1 and mixed well. After reacting for 30 min at room temperature, the absorbance (A) was measured at 517 nm. Ascorbic acid (1–100 μg/ml) and anthocyanin standards (0.1–10 μg/ml) were used as positive controls, and a DPPH solution without a sample was used as a negative control. The IC_50_ value, which represents the concentration at which 50% of DPPH oxygen radicals were inhibited, was determined. The DPPH clearance rate was calculated by the following formula:

$$\text{Inhibition}\left(\%\right)=\frac{\left[\text{Ac}-\text{Acb}\right]-\left[\text{As}-\text{Asb}\right]}{\left[\text{Ac}-\text{Acb}\right]}\times 100.$$

**Table 1:** The experimental design for assay of scavenging DPPH

	A_C_	A_CB_	A_S_	A_SB_
Sample solution(ml)	0	0	2.0	2.0
DPPH solution(ml)	2.0	0	2.0	0
Distilled water (ml)	2.0	4.0	0	2.0

Note

A_C_: sample free, with DPPH. A_CB_: sample free, DPPH free. A_S_: with sample and DPPH. A_SB_: with sample, DPPH free.

#### 2.6.3 Superoxide anion scavenging activity

This assay was performed according to the method described in Zhang's paper (Zhang & Yang, 2005). The following configuration solution was used (solvent is distilled water): pH 8.5; 50 mmol/L Tris‐HCl buffer; 1 mmol/L EDTA solution; 0.4 mmol/L PR solution; and 100 mmol/L DTT solution. Samples were prepared in distilled water at different concentrations (1–100 μg/ml). The reagent and sample were added to the test tube in accordance with Table 2 and mixed well. After allowing the reaction to progress for exactly 10 min, 30 μl of DTT solution was added to terminate the reaction. The absorbance (A) was measured at 325 nm 1 hr after terminating the reaction. Ascorbic acid (1–100 μg/ml) and anthocyanin standards (0.1–10 μg/ml) were used as positive controls. The O_2_^−^ clearance rate was calculated by the following formula:

$$\text{Inhibition}\left(\%\right)=\frac{\left[\text{Ac}-\text{Acb}\right]-\left[\text{As}-\text{Asb}\right]}{\left[\text{Ac}-\text{Acb}\right]}\times 100.$$

**Table 2:** The experimental design for assay of scavenging O_2_^−^

	A_C_	A_CB_	A_S_	A_SB_
Tris‐HCl buffer (ml)	1.0	1.0	1.0	1.0
EDTA solution(ml)	1.0	1.0	1.0	1.0
Distilled water (ml)	0.4	1.4	0	1.0
Sample solution(ml)	0	0	0.4	0.4
Pyrogallol solution (ml)	1.0	0	1.0	0

Note

A_C_: sample free, with PR. A_CB_: sample free, PR free. A_S_: with sample and PR. A_SB_: with sample, PR free.

### 2.7 Determination of tyrosinase inhibitory activity

The tyrosinase inhibition test of red rice bran anthocyanins was based on the study by Liu, Jiao, and Zhang (2013). The reagents listed in Table 3 were added in sequence and reacted in a 37°C water bath for 15 min. A total of 100 μl of the supernatant was added to a 96‐well plate, and the absorbance of each sample solution was measured at 475 nm. Different concentrations of ascorbic acid solution were used as a positive control. The inhibition rate of tyrosinase by each concentration of the samples was calculated by the following formula:

$$\text{Inhibition}\left(\%\right)=\frac{\left[\text{Ac}-\text{Acb}\right]-\left[\text{As}-\text{Asb}\right]}{\left[\text{Ac}-\text{Acb}\right]}\times 100.$$

**Table 3:** The experimental design for assay of tyrosinase inhibition

	A_C_	A_CB_	A_S_	A_SB_
Phosphate buffer (ml)	0.8	0.9	0.6	0.7
Sample solution (ml)	0	0	0.2	0.2
Tyrosinase solution (ml)	0.1	0	0.1	0
L‐Dopa (ml)	0.1	0.1	0.1	0.1

Note

A_C_: sample free, with TYR. A_CB_: sample free, TYR free. A_S_: with sample and TYR. A_SB_: with sample, TYR free.

### 2.8 Statistical analysis

Data are expressed as the mean ± standard deviation of three replicates. Data analysis was performed using Excel 2016 and SPSS statistics version 17, and RSM optimization analysis was performed using Design Expert 8.0.6. *p* value of <.05 was considered statistically significant by Student *t* test or ANOVA followed by Duncan's analysis test.

## 3 RESULT AND DISCUSSION

### 3.1 The anthocyanins contents

**Figure 2:**
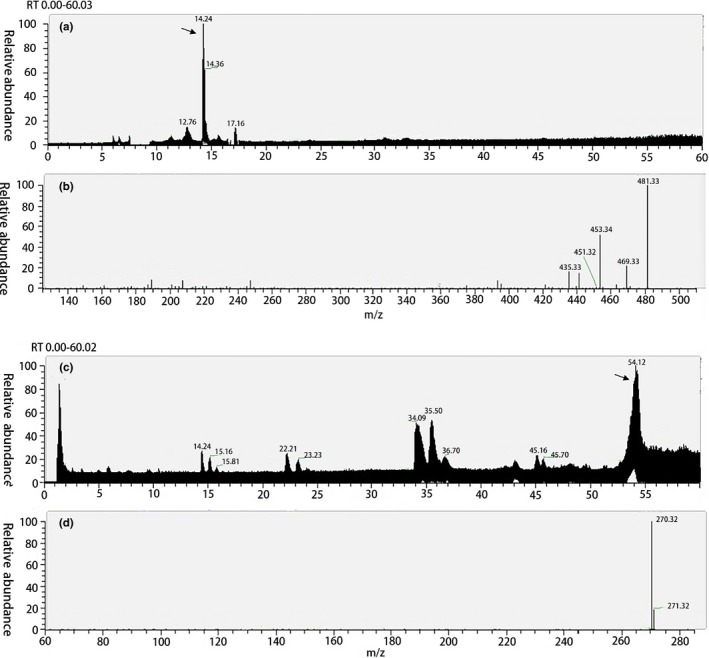
The total ion chromatogram of alcohol extract (a) and water extract (c); fragment ion peak of alcohol extract (b) and water extract (d)

The anthocyanin extract obtained in this experiment was performed by HPLC/MS. As shown in Figure 2a,b, the main component of the alcohol extract is mainly concentrated in 14 min, and the particle fragment *m/z* is 481.33 [M + H]^+^. Compared with the standard substance, this compound is paeoniflorin (*m/z* = 480). Unlike the alcohol extract, the main component of the water extract is concentrated at 54.12, and its main fragment ion peak is 270.32 (Figure 2c,d). Although water has many advantages as an extractant, the presence of water‐soluble nutrients reduces the extraction concentration and purity of anthocyanins. Therefore, most of the anthocyanin extraction uses ethanol as an extractant (Hosoda et al., 2018). Consistent with the previous report, the paeoniflorin was main substance in colored rice, especially black rice and red rice (Abdel‐Aal et al., 2006; Pedro, Granato, & Rosso, 2015).

### 3.2 Absorption spectrum of red rice bran anthocyanins

**Figure 3:**
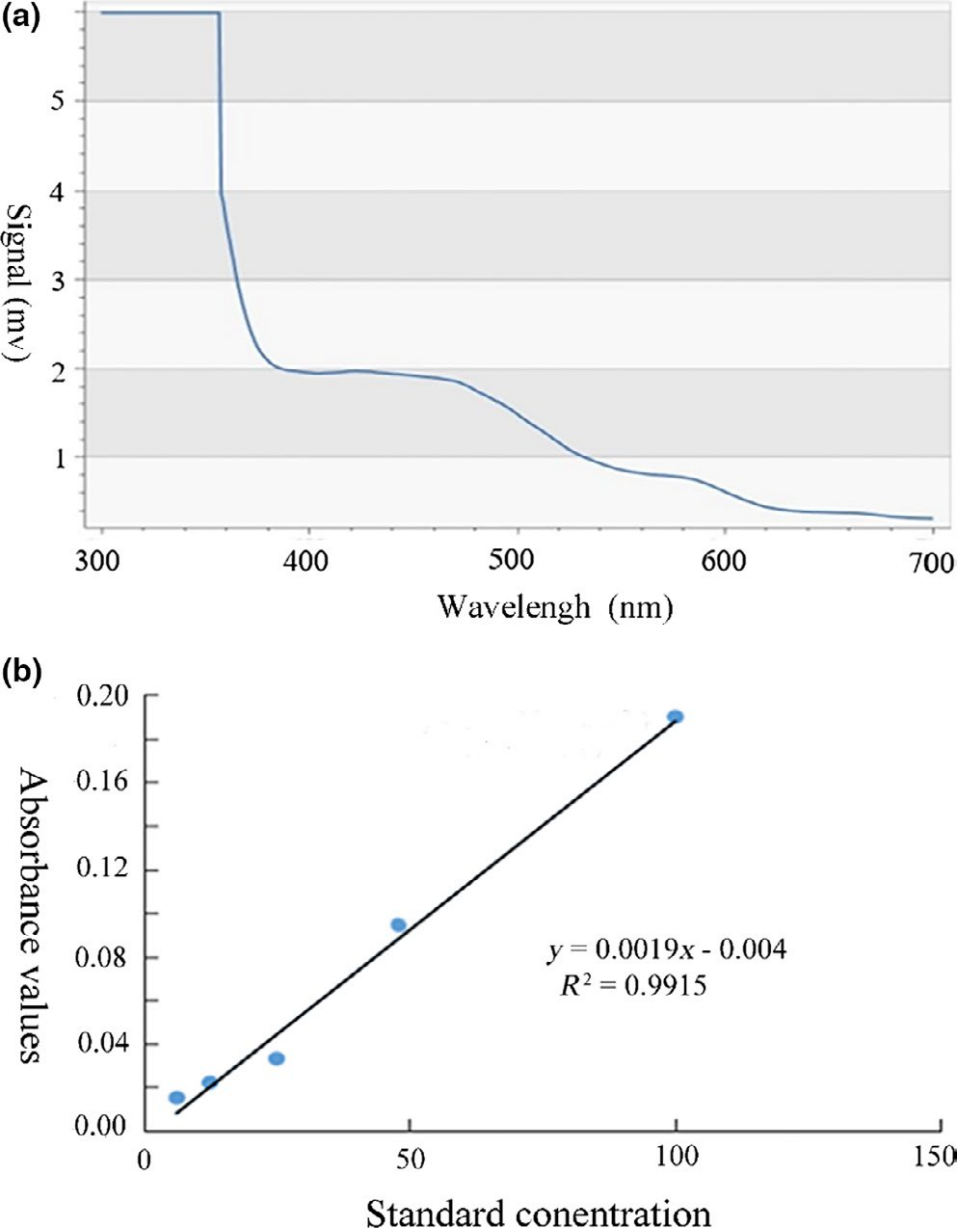
(a) Absorption spectrum of red rice bran anthocyanins. (b) The standard curve of paeoniflorin

As Figure 3a shows that the maximum absorption wavelength of the red rice bran anthocyanins was 440 nm. However, the absorption wavelengths of the common anthocyanins are between 450 and 550 nm, probably because the parental structure of the anthocyanins in red rice bran is slightly different from that of other common flavonoids. An anthocyanin‐like structure in which the hydroxyl group at position 5 of the core structure of the pigment is unsubstituted would shift the maximum absorption wavelength to a blue color (Pedro et al., 2015) and result in a decrease in the maximum absorption wavelength of the anthocyanin in red rice bran.

### 3.3 Red rice bran anthocyanin standard curve

The standard curve of paeoniflorin was also plotted. As shown in Figure 3b, the regression equation of the concentration of paeoniflorin and the absorbance value was *y* = 0.0019 × −0.004 μg/ml, with *R* ^2^ = 99.15%. This result indicated that within a certain range, the linear relationship between the concentration and absorbance of paeoniflorin was good. Therefore, this standard can accurately reflect the relationship between the absorbance value and the concentration of the anthocyanins in red rice bran

### 3.4 Response surface experiment results

#### 3.4.1 Selection of extraction parameters

**Figure 4:**
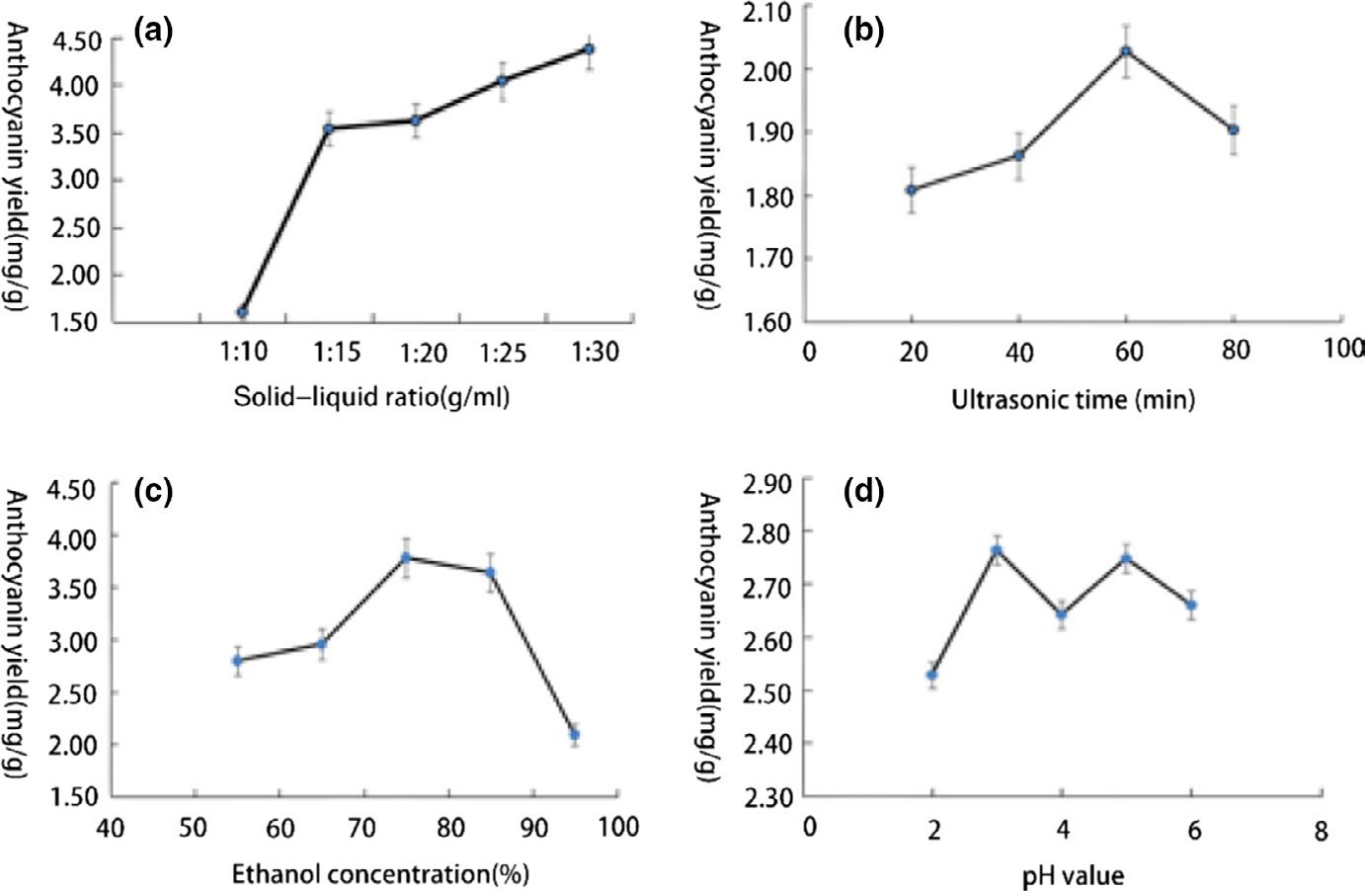
(a) The effect of solid–liquid ratio on the yield of anthocyanin in red rice bran. (b) The effect of ultrasonic time on the yield of anthocyanin in red rice bran. (c) The effect of ethanol concentration on the yield of anthocyanin in red rice bran. (d) The effect of acidity on the yield of anthocyanin in red rice bran

The process of extracting the chemical constituents from the plant raw materials is affected by multiple factors. The results of single‐factor experiments show that the optimum conditions for extracting anthocyanins are a ratio of material to liquid of 1:15, 60 min of sonication, a 75% ethanol concentration, and a pH of 3 (Figure 4). During ultrasonic extraction, the temperature of the samples increased and some of the anthocyanin activity is destroyed due to the excessive temperature. To save costs and avoid wasting reagents, a 75% ethanol concentration was chosen. The actual values and coded values for the variables are summarized in Table 4.

**Table 4:** Coded values of variables in the study

Variable	Nomenclature		Value		
			(−1)	0	(1)
Solid–liquid ratio	*X*_1_	(g/ml)	1:10	1:15	1:20
Ethanol concentration	*X*_2_	(%)	65	75	85
Ultrasonic time	*X*_3_	(min)	40	60	80
pH value	*X*_4_		2	3	4

A Box‐Behnken design was used to evaluate the effects of four variables (solid–liquid ratio, ethanol concentration, ultrasonication time, and pH value) on the ultrasound‐assisted extraction of anthocyanins. The purpose was to determine the best extraction process for extracting anthocyanins from red rice bran. In this experiment, the yield of red rice bran anthocyanins was the response index. The results are shown in Table 5.

**Table 5:** Response surface experimental design and results

Run	Solid–liquid ratio (g/ml)	Ethanol (%)	Time (min)	pH	Anthocyanins yield (mg/g)
1	−1	1	0	−1	2.70
2	−1	0	0	1	2.69
3	−1	0	0	1	2.61
4	1	0	0	0	4.89
5	−1	0	−1	1	2.52
6	1	0	−1	0	4.78
7	−1	0	1	0	2.69
8	0	0	−1	−1	4.94
9	0	0	−1	0	4.79
10	0	1	−1	1	3.47
11	0	1	1	−1	3.79
12	0	1	0	0	4.51
13	0	1	0	0	4.45
14	1	1	0	0	4.91
15	0	0	1	0	4.07
16	0	−1	−1	0	4.12
17	0	−1	1	−1	4.49
18	0	−1	0	−1	4.02
19	0	−1	0	0	4.11
20	0	0	0	0	5.69
21	0	0	0	0	5.59
22	0	0	0	0	5.59
23	0	0	0	0	5.87
24	−1	−1	0	0	3.46
25	1	−1	0	0	5.17
26	0	0	1	0	4.71
27	1	0	0	1	4.39
28	1	0	1	−1	4.46
29	0	0	0	1	5.16

#### 3.4.2 Analysis of variance in the extraction rate of the red rice anthocyanins

**Figure 5:**
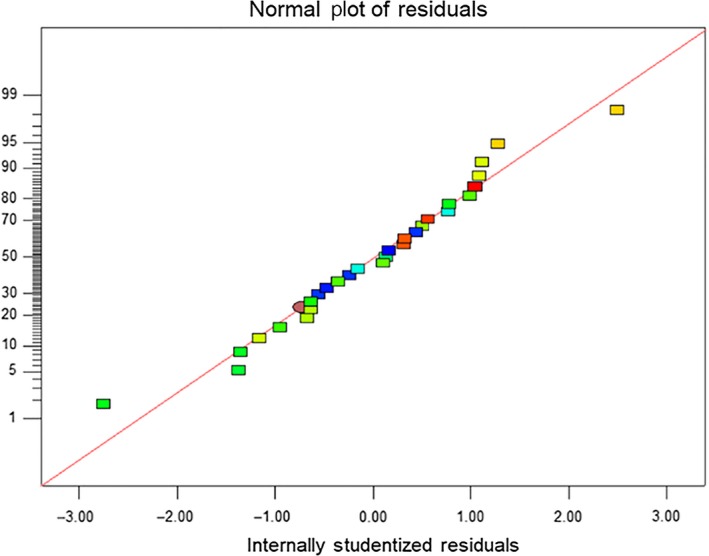
Residual model diagram

The variance analysis results of model Y are shown in Table 6. The regression model was extremely significant (*p* ˂ .01). The determination coefficient (*R* ^2^) is an indicator for evaluating the fitting effect of the regression model; the *R* ^2^ of this regression model was 0.9045, indicating that the fitting effect was good. The normal plot of the residuals is shown in Figure 5. Most of the residuals are normally distributed, and the data points are quite close to the fitted line. These observations indicated that the miscalculation was not significant. According to the *F* value shown in Table 6, the solid–liquid ratio was found to be the most significant factor affecting the anthocyanin yield, followed by the ethanol concentration, pH value, and ultrasonic extraction time. The quadratic terms were not significant (*p* ˃ .05).

**Table 6:** The variance analysis results of the quadratic regression model

Source	Sum of squares	Degree of freedom	Mean square	*F* value	*P* value	Significant
Model	24.18	14	1.73	9.47	˂.0001	**
*X*_1_–*X*_1_	6.54	1	6.54	35.84	˂.0001	**
*X*_2_–*X*_2_	0.87	1	0.87	4.20	.0623	
*X*_3_–*X*_3_	0.44	1	0.44	2.43	.1416	
*X*_4_–*X*_4_	0.67	1	0.67	3.70	.0751	
*X*_1_ *X*_2_	0.23	1	0.23	1.24	.2834	
*X*_1_ *X*_3_	7.51E‐03	1	7.51E‐03	0.04	.8421	
*X*_1_ *X*_4_	2.23E‐03	1	2.23E‐03	0.01	.9134	
*X*_2_ *X*_3_	0.48	1	0.48	2.63	.1273	
*X*_2_ *X*_4_	0.39	1	0.39	2.13	.1661	
*X*_3_ *X*_4_	0.10	1	0.10	0.55	.4700	
*X*_1_^2^	4.72	1	4.72	25.90	.0002	
*X*_2_^2^	1.82	1	1.82	9.96	.0070	
*X*_3_^2^	1.78	1	1.78	9.79	.0074	
*X*_4_^2^	0.60	1	0.60	3.28	.0915	
Total error	2.55	14	0.18			
Lack of fit	2.26	8	0.28	5.80	.0530	
Pure error	0.29	6	0.05			
Total SS	26.73	28				

Note

Predicted *R* ^2^ = .9045 CV = 9.95%.

#### 3.4.3 Response surface interaction analysis

**Figure 6:**
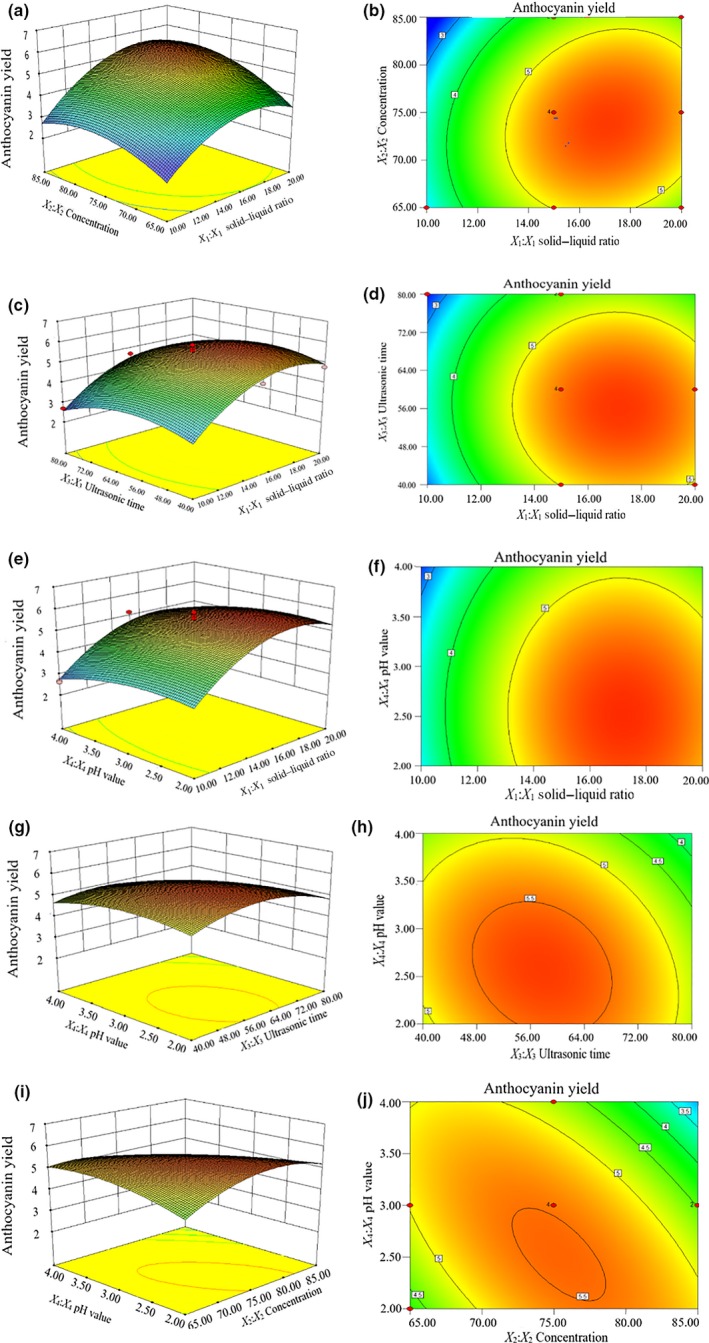
Response surface and contour plots for the effects of the interactions of the solid–liquid ratio and ethanol concentration (a and b), solid–liquid ratio and ultrasonic time (c and d), solid–liquid ratio and pH (e and f), ultrasonic time and pH (g and h), and pH and ethanol concentration (i and j) on the yield of anthocyanin from red rice bran

The response surface curves shown in Figure 6 reveal that the curve was steep. This result indicates that the response surface value fluctuates greatly. The interaction of the solid–liquid ratio, ethanol concentration, ultrasonication time, and pH on the red rice bran anthocyanin yield was relatively large. However, the contours of the interaction were round. The results were consistent with the analysis of the variance showing that the interaction was not significant.

Figure 6a,b shows that when the ultrasonication time and pH are set to zero and the ethanol concentration is fixed, the anthocyanin yield of red rice bran tends to increase as the solid–liquid ratio increases. Specifically, when the solid–liquid ratio was 1:15.81, the anthocyanin yield reached the maximum value. When the solid–liquid ratio was fixed, the yield first increased with the increasing ethanol concentration and then decreased. The highest anthocyanin yield was with 75% ethanol. Combined with the density degree of the contour line, the effect of the solid–liquid ratio on the anthocyanin yield was greater than that of the ethanol concentration.

Figure 6i,j shows the response surface plot of the effects of the pH and ethanol concentration. The interaction between these two variables is mainly impacted by the ethanol concentration. Additionally, in Figure 5g,h, combined with the density degree of the contour line, the effect of the pH on the anthocyanin yield is greater than that of the ultrasonic extraction time.

In summary, the relative impacts of the variables on the anthocyanin yield of red rice bran are in the order material–liquid ratio > concentration >pH > ultrasonication time, which is consistent with the analysis of variance. Our results were in good agreement with the studies by Cai and Luo (Shun‐Jing et al., 2016).

#### 3.4.4 Verification experiment

The optimal extraction conditions of the red rice bran anthocyanins obtained from the response surface were a material–liquid ratio of 1:17.46, ethanol concentration of 78.37%, extract pH of 2.31, and ultrasonication time of 55.23 min. The predicted red color obtained under these conditions was obtained. The yield of rice bran anthocyanins was 5.799 mg/g.

The above optimal conditions were adjusted to the following in the actual extraction of red rice bran anthocyanins: a material–liquid ratio of 1:17; ethanol concentration of 78%; extract pH of 2.3; and ultrasonication time of 55 min. The extraction yield was 5.512 mg/g, which was close to the theoretical prediction. Therefore, the optimized conditions for the extraction of red rice bran anthocyanin obtained by RCM have practical application value.

Anthocyanins have high solubility in water and alcohol. The purpose of selecting an aqueous ethanol solution is not in solubility but in purity. Water‐soluble polysaccharides, proteins, peptides, soluble starch, and other nutrients are also in the bran (Xue, Zhang, Zhang, & Ren, 2019). At the same time as water extraction of anthocyanins, the above substances have to be obtained. The content of such substances is much higher than the content of anthocyanins. Therefore, most of the anthocyanin extraction will use the ethanol–water system (Celli, Ghanem, & Brooks, 2015; Das, Goud, & Das, 2016; Haiwei, 2015).

Consistent with most anthocyanin extraction, the parameters obtained by the response surface method are similar to the results of this test (Wen et al., 2015). Temperature is an important parameter, and the optimum extraction temperature is room temperature (Chen, Choi, et al., 2012). Because the content of anthocyanins in black rice may be higher than that of red rice, most research studies on colored rice focus on black rice (Chaiyasut et al., 2017; Khazaei, Jafari, Ghorbani, Kakhki, & Sarfarazi, 2016). Supercritical extraction and artificial neural methods are used in the extraction of rice bran extract, which may be a direction for later development (Das et al., 2016; Sookwong et al., 2016).

### 3.5 Antioxidant capacity

**Figure 7:**
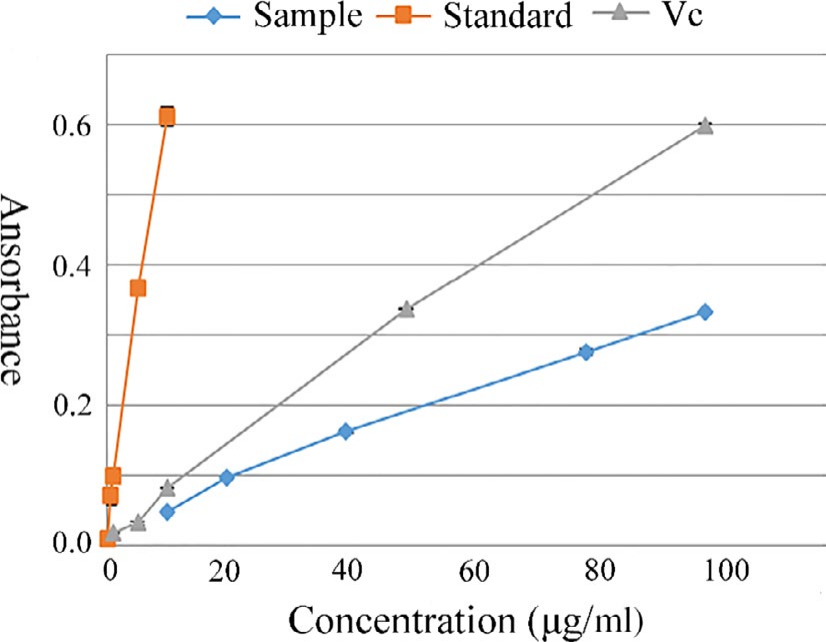
The reducing of anthocyanin sample, standard, and vitamin C

Vitamin C (Vc) has strong antioxidant activity and is used as a positive control (Lee, Han, Kim, Baek, & Baik, 2013). As mentioned in the methods, a high absorbance value reflects a strong reduction capacity. As shown in Figure 7, the anthocyanin standard resulted in the highest reduction, followed by Vc, and finally the anthocyanin extracted from red rice bran. Table 7 presents the IC_50_ values of the anthocyanins extracted from red rice bran, the anthocyanin standards, and Vc against DPPH. The anthocyanin standard has a stronger ability to scavenge DPPH than does Vc (*p* < .05). The DPPH scavenging ability of anthocyanins extracted from red rice bran did not reach the level of the standards (*p* < .05). The data in Table 7 clearly show the weak ability of the anthocyanin extracted from red rice bran to scavenge oxygen free radicals (˃2 mg/ml). A variety of methods have been used to measure the antioxidant activity of colored rice extracts (Abdel‐Aal et al., 2008; Shen et al., 2015). Comparison studies have shown that the red rice extract has an excellent iron‐reducing ability and moderate free radical scavenging activity. The excellent antioxidant activity of red rice is attributed to the presence of anthocyanins (Cai, Sun, Xing, Luo, & Corke, 2006). Plant polyphenols, including anthocyanins, have an in vitro antioxidant activity that depends on the chemical structure of the polyphenols; for example, the aglycone structure and the attached sugar moiety result in free radical scavenging activity against O_2_^−^ and ONOO^‐^. The antioxidant activity of the red rice extract may be affected by the origin and maturity of the rice. The former is less pure than the latter may be another reason why the extract is less active than the standard.

**Table 7:** IC_50_ values of anthocyanin sample, standard and vitamin C (μg/ml)

	DPPH radical scavenging activity	Superoxide anion scavenging activity	Tyrosinase inhibition
Anthocyanin sample	53.51 ± 1.69	2,375 ± 35.46	4.26 ± 0.59
Anthocyanin standard	14.60 ± 0.89	64.74 ± 1.06	2.60 ± 0.16
vitamin C	24.45 ± 1.57	136.25 ± 2.86	2.18 ± 0.42

Although preliminary experiments have been found that colored rice has better antioxidant activity than alcohol extract of white rice (Nam et al., 2006), the antioxidant properties of nutrients in rice bran mainly come from phenolic substances (Chen, Choi, et al., 2012; Laokuldilok, Shoemaker, Jongkaewwattana, & Tulyathan, 2011). This does not affect these colored plants which could provide a potential natural source of anthocyanins and could be beneficial to the human health, especially in the neurodegenerative disorders and as sources of natural antioxidants in food industry. However, anthocyanins are easily degraded, resulting in darker colors, resulting in reduced product quality (Ibemhal, Imotomba, Mazumder, & Laishram, 2015; Tananuwong & Tewaruth, 2010).

### 3.6 Tyrosinase inhibitory activity

Tyrosinase plays a key role in melanin production and browning, and many plant extracts have been found to inhibit tyrosinase activity, such as jackfruit and dandelion (Suh, Hwang, Park, Park, & Lee, 2014). Plant extracts have become a natural source of tyrosinase inhibitors (Bonesi et al., 2019). Vc, which has a strong inhibitory effect on tyrosinase, was used as a control. Vc binds to the Cu^2+^ in tyrosinase, preventing the activation of the tyrosinase and thereby inhibiting the tyrosinase. Low concentrations of red rice bran anthocyanins can inhibit the activity of tyrosinase. The extent of the tyrosinase inhibition provided by the red rice bran anthocyanins and Vc increased gradually and finally stabilized. The inhibitory effect of Vc was better than that of the red rice bran anthocyanins (*p* < .05). The lower the IC_50_ value is, the stronger the biological activity. Therefore, red rice bran anthocyanin has a good inhibitory effect on tyrosinase, but the inhibition provided by these anthocyanins is slightly weaker than that provided by Vc.

Anthocyanin contents (3G [2.84 mg/g], D3G [0.34 mg/g], and P3G [0.35 mg/g]) from the seed coat of black soybean possessed antihuman tyrosinase activity (Jhan et al., 2016). Anthocyanins in cranberry (*Vaccinium macrocarpon*) and blueberry (*Vaccinium myrtillus*) are the most potential for tyrosinase inhibitors and can be developed as whitening agent (Cásedas, Les, Gómez‐Serranillos, Smith, & López, 2017).

## 4 CONCLUSION

Response surface methodology was used to evaluate the effect of temperature, time, and solid–liquid ratio on the extraction of compounds from red rice bran. The optimal extraction conditions were a material–liquid ratio of 1:17.46, ethanol concentration of 78.37%, extract pH of 2.31, and ultrasonication time of 55.23 min. The predicted yield of red rice bran anthocyanins obtained under the optimal conditions was 5.799 mg/g, and the actual yield of red rice bran was 5.51 mg/g, which is not much different from the expected value. The use of acid–ethanol is noteworthy because it is a nontoxic solvent system which could be used to extract bioactive and pigmented compounds from black rice, and also can reduce the content and type of impurities in the extract, which is beneficial to industrial production.

In this experiment, paeoniflorin was the main anthocyanin identified in red rice bran. The extract had good antioxidant and tyrosinase inhibition activities. There is a good correlation between the observed effect and concentration. Therefore, the anthocyanins extracted from red rice bran can be applied to the health food, medical, and beauty fields. Moreover, these results can not only improve the utilization rate of red rice bran but also provide new ideas for the development of natural pigments.

## CONFLICT OF INTERESTS

No conflict of interest was declared by the authors.

## ETHICAL APPROVAL

This study does not involve any human or animal testing.
